# Microbiota and Ocular Diseases

**DOI:** 10.3389/fcimb.2021.759333

**Published:** 2021-10-21

**Authors:** Wei Xue, Jing Jing Li, Yanli Zou, Bin Zou, Lai Wei

**Affiliations:** ^1^ State Key Laboratory of Ophthalmology, Sun Yat-sen University, Guangzhou, China; ^2^ Department of Ophthalmology, Affiliated Foshan Hospital, Southern Medical University, Foshan, China

**Keywords:** gut microbiome, gut-eye axis, ophthalmic diseases, multiomics, microbial therapeutics

## Abstract

Recent advances have identified significant associations between the composition and function of the gut microbiota and various disorders in organ systems other than the digestive tract. Utilizing next-generation sequencing and multiomics approaches, the microbial community that possibly impacts ocular disease has been identified. This review provides an overview of the literature on approaches to microbiota analysis and the roles of commensal microbes in ophthalmic diseases, including autoimmune uveitis, age-related macular degeneration, glaucoma, and other ocular disorders. In addition, this review discusses the hypothesis of the “gut-eye axis” and evaluates the therapeutic potential of targeting commensal microbiota to alleviate ocular inflammation.

## 1 Introduction

Dysbiosis in the gut microbiota has been linked to multiple gastrointestinal disorders, such as inflammatory bowel disease, irritable bowel syndrome, and celiac disease (Rajilić-Stojanović et al., 2015; Bernstein and Forbes, 2017; Chibbar and Dieleman, 2019). Recent decades have seen ample evidence documenting a much broader stimulatory/regulatory role of the gut microbiota in disorders involving organ systems other than the digestive tract (Jangi et al., 2016; Scher et al., 2016).

The eye and brain are two organs remote from the intestines and considered unrelated to any effects exerted by the gut microbiota. However, the emergence of microbiota as a crucial regulator of brain function has led to increasing attention to the communication that occurs between the gut microbiota and the central nervous system (Cryan et al., 2019). Eye-related diseases and their associations with the gut microbiome are the focus of the current review. The gut is occupied by trillions of microbes and thousands of species suitable for high-throughput sequencing technologies. Utilizing next-generation sequencing and multiomics approaches, the microbial community that possibly impacts ocular disease has been identified. Here, we review the common methods used to study the microbiome, with a particular focus on several meta-approaches and animal models. We also discuss the “gut-eye axis” and the interrelationship between intestinal/ocular microbiota and several ophthalmic diseases, such as autoimmune uveitis, age-related macular degeneration (AMD), glaucoma, and other ocular disorders. Finally, the therapeutic potential of targeting commensal microbiota is evaluated.

## 2 Influences of the Gut Microbiome and Analysis Approaches

### 2.1 Gut Microbiome

The gut microbiome has well-defined roles in digestion, production of vitamins, synthesis of short-chain fatty acids (SCFAs) (such as acetate, propionate, and butyrate), protection against pathogenic bacteria, and development of the host immune system. In addition, the gut microbiome helps preserve the homeostasis of several T-cell populations in the gut, comprising regulatory T cells (Tregs), T helper 1 (Th1) cells and 17 (Th17) cells, which are vital in hosting an immune response against pathogens (Kho and Lal, 2018). These effects might be mediated by the microbes themselves or indirectly by their metabolites. For example, the SCFAs produced by some intestinal microbes, such as *Bacteroides*, can regulate Treg and Th17 cells in the intestine, circulation, and extraintestinal tissues (Zeng and Chi, 2015).

The human gut microbiota is composed of 6 main phyla: *Firmicutes*, *Bacteroidetes*, *Actinobacteria*, *Fusobacteria*, *Verrucomicrobia*, and *Proteobacteria* (The Human Microbiome Project Consortium, 2012). Of these, *Firmicutes* and *Bacteroidetes* represent 70–90% of the gut microbiota (Kho and Lal, 2018). In a healthy human, the gut bacterial microbiome maintains a delicate balance between “good or beneficial” (probiotic and anti-inflammatory) and “bad or harmful” (pro-inflammatory and pathogenic) bacteria. Increases in the abundance of proinflammatory bacteria, *e.g.*, *Escherichia coli*, and decreases in the abundance of anti-inflammatory bacteria, *e.g.*, *Faecalibacterium prausnitzii*, are closely related to autoimmune intestinal diseases, including Crohn’s disease and ulcerative colitis (Hornef and Pabst, 2016; Viladomiu et al., 2017). Disturbances in the gut microbiota have been implicated in a wide range of diseases, including irritable bowel syndrome (Rajilić-Stojanović et al., 2015), inflammatory bowel disease (Sheehan et al., 2015), obesity (Aron-Wisnewsky and Clément, 2016), diabetes (Karlsson et al., 2013; Forslund et al., 2015; Tai et al., 2015), multiple sclerosis (Jangi et al., 2016), rheumatoid arthritis (Scher et al., 2016), graft-versus-host disease (Mathewson et al., 2016), and neurodegenerative diseases (Ghaisas et al., 2016), many of which are associated with serious ophthalmic sequelae.

### 2.2 Approaches to Microbiome Analysis

A crucial step in traditional microbiological studies is the isolation of microorganisms from samples while avoiding contamination from human handling as much as possible. There are multiple options for modern “-omics” studies. Here, we discuss several analytical approaches to microbiome studies in the context of ocular diseases.

Traditionally, isolation and direct culture have been used for microbial investigations, and these strategies are still used today. However, culture-based techniques can only be used to characterize a small percentage of the actual microbial populations in a sample because they are limited by the phenotypic characteristics of microbes, for example, the ability of microbes in a sample to proliferate in or on a specified growth medium under a specified growth condition (Schabereiter-Gurtner et al., 2001; Hori et al., 2008; Fernández-Rubio et al., 2010; Ozkan et al., 2017). Meantime, combining quantitative PCR with negative staining transmission electron microscopy is an effective means of identifying microbial populations (Deng et al., 2021). In fact, cultivated bacteria represent only half of the bacterial phyla thus far Schloss (Schloss and Handelsman, 2004)

Given the widespread use of next-generation sequencing technologies, the high-throughput analysis of microbial communities has become a much lower cost and less time-consuming approach that allows researchers to achieve a more complete understanding of the composition of commensal communities. Since it is difficult to study underrepresented bacteria, such as those of the eye, by traditional methods, this advance has been especially crucial for studying ocular communities (Stein-Streilein, 2008; Cone and Pais, 2009). 16S/18S/ITS gene amplicons and shotgun sequencing are usually used to analyze microbial populations (Clarridge, 2004; Rausch et al., 2019).

#### 2.2.1 Multiomics Approach

The multiomics approach has been applied to sort out the molecular interactions in cells and tissues in complex disease settings. Here, we broadly classify the multiomics approach into metagenomics, metatranscriptomics, metaproteomics, and metametabolomics and discuss recent discoveries in ocular diseases utilizing these approaches (**Table 1**).

**Table 1:** Advantages and disadvantages of the multiomics approaches.

Multiomics approach	Advantages and Disadvantages	Literature	
Metagenomics	Reveals in revealing the low abundant cultivable and non-cultivable microorganisms in a given sample	Abundant microbial species increased in BD and VKHD patients	(Ye et al., 2018; Ye et al., 2020)
	Biases in amplifications due to equal affinities of primers in marker gene analysis	Identifying the intraocular microbiota signature in glaucoma and AMD	(Deng et al., 2021)
	Inconformity of taxonomic abundance and transcriptional activities	*Veillonella* as the predominant genus in patients of chronic dacryocystitis	(Eguchi et al., 2017a)
	Lack of abundance of gene functional annotation database	The abundance of *bacillus* and *Enterococcus faecium* in MGD	(Zhao et al., 2020)
		Microbiological differences between conjunctival swabs and lid	(Zysset-Burri et al., 2021)
Metatranscriptomics	Provides high-throughput microbial transcriptome information of cultivable and non-cultivable species	Glucorhamnan from *Ruminococcus gnavus* induces dendritic cells to secret TNFα in with Crohn’s disease	(Henke et al., 2019)
	rRNA contamination		
	No standard methods for RNA acquisition	*Ruminococcus gnavus* regulars intestinal barrier function	(Graziani et al., 2016; Luissint et al., 2016)
	Differences in ribosome binding ability to mRNA leads to affect the analysis of the functional protein	Basic metabolism are severely impaired in the duodenal microbiota of obese patients	(Granata et al., 2020)
Metaproteomics	Allows for large-scale identification and quantification of microbial proteins, and reveals the function and metabolic pathway of the microbiota	Phylogenetic changes in bacteria in intestinal inflammation	(Mayers et al., 2017)
	Contamination from host and undigested proteins		
	No standardized method for protein preparation		
	Lack of the protein database for identification		
Metametabolomics	Creates profiles of microbial metabolites and reveals metabolic patterns of the microbiota	Biomarkers of distinguishing AAU progression and treatment response	(Guo et al., 2014; Luissint et al., 2016)
	No standardized method for metabolites preparation		
	The uncertain origin of the microbial metabolites	Detrimental co-metabolites of determining specific bacterial genomes in genetic and simple obesity in children	(Heintz-Buschart et al., 2016)

##### 2.2.1.1 Metagenomics

Metagenomics is used to sequence the genomes of microbes, *e.g.*, archaea, bacteria, viruses, fungi, parasites and their entire functional profiles, present in a given sample (Lorenzon et al., 2018; Almeida et al., 2019; Coker et al., 2020; Daliri et al., 2020; Gregory et al., 2020; Nagpal et al., 2020). Two approaches are commonly used, namely, marker gene analysis and shotgun sequencing. These two methods allow for the analysis of genes of interest and the analysis of detailed genetic and taxonomic information, respectively. Metagenomic profiles have been characterized in Behcet’s disease (BD) (Ye et al., 2018) and Vogt-Koyanagi-Harada disease (VKHD) patients (Ye et al., 2020). These two groups of patients show distinctly abundant microbial species compared with controls. Interestingly, by using metagenomic sequencing, our group provided preliminary evidence that the intraocular microbiota signature might also be disease-specific in ocular diseases such as glaucoma and AMD (Deng et al., 2021). *Veillonella* have been found as the predominant genus in patients of chronic dacryocystitis through metagenomic sequencing (Eguchi et al., 2017a).

Shotgun sequencing profiles may present information about the potential function(s) of an entire microbial community at the gene level based on databases of microbial genomes with known sequences, yet the information yielded by this method may not accurately represent what is happening in the gut at a given time (Emerson et al., 2017). This is supported by studies of taxonomic abundance that do not correspond to transcriptional activities (Abu-Ali et al., 2018; Jia et al., 2019; Granata et al., 2020). Utilizing this method, distinct meibum microbial communities were identified in patients with meibomian gland dysfunction and in healthy controls, indicating different immune evasive virulence of the meibum microbiota (Zhao et al., 2020). The ocular surface microbiome identified by metagenome shotgun sequencing have recently been associated with the tear proteome, suggesting a role in human immune defense (Zysset-Burri et al., 2021).

##### 2.2.1.2 Metatranscriptomics

Metatranscriptomics exploits RNA sequences to reveal the presence and quantity of microbial RNA in a biological sample at a given time. Shotgun metatranscriptomics can answer the question of what microbes are doing “right then” in an ecosystem (Franzosa et al., 2014). Analyzing their RNA provides clues to determining their metabolic activities in response to stress and how such alteration in metabolic activity influences host phenotypes in healthy or diseased conditions (Daliri et al., 2021).

*Ruminococcus gnavus*, a prevalent member of the human gut microbes, has been found to produce an inflammatory polysaccharide, i.e., glucorhamnan, which induces dendritic cells to secrete TNFα in patients with Crohn’s disease (Henke et al., 2019). The same bacterial species has also been reported to fortify gut barrier functions by modulating mucin production in the gut, which may eventually prevent gut mucosal inflammation (Graziani et al., 2016; Luissint et al., 2016). Using metatranscriptomics, Granata et al. (2020) showed that carbohydrate, amino acid, and nucleotide metabolism are severely impaired in the duodenal microbiota of obese patients compared to nonobese patients.

##### 2.2.1.3 Metaproteomics

Metaproteomics employs high-resolution mass spectrometry to identify and measure the levels of expressed proteins (Mesuere et al., 2016). A microbial protein may have direct or indirect effects on other microbes and influence host physiology (Schweppe et al., 2015; Rolig et al., 2018), and identifying and quantifying gut microbial proteins (metaproteomics) provide a better picture of the role of gut microbes in health and disease. The information obtained is processed by pipelines that eventually match the peptides with metagenomic databases to determine the most likely microbes that might have expressed the proteins. The ability of metaproteomics to identify and quantify proteins from microbial and host sources makes it a powerful approach in studying microbial–host interactions (Gavin et al., 2018). Several platforms, such as MetaLab (Cheng et al., 2017), MetaQuantome (Easterly et al., 2019), Galaxy-P (Jagtap et al., 2015), and MetaProteomeAnalyzer (Muth et al., 2018), are available for processing metaproteomic data. Metaproteomics enables a clear identification of how disease conditions (or their triggers) result in gut microbial dysbiosis and which microbial protein may directly affect the host (Zhang X. et al., 2018; Long et al., 2020). For example, quantitative alterations in both host and microbial proteins due to intestinal inflammation can be highlighted *via* metaproteomics, and this approach can corroborate observed phylogenetic changes in bacteria (Mayers et al., 2017).

##### 2.2.1.4 Metametabolomics

Metabolomics involves the study of metabolites in a biological sample as well as the exploration of microbe-derived therapeutic products. A targeted or untargeted approach can be used (Daliri et al., 2021). The former is used to study metabolites in specific pathways associated with a particular disease, while the latter measures as many metabolites in the sample as possible (Commisso et al., 2013; Vignoli et al., 2019). Analytical techniques include liquid chromatography, gas chromatography, mass spectrometry (MS), MS/MS, ultraviolet/visible spectroscopy and nuclear magnetic resonance spectroscopy (Daliri et al., 2017). Metabolomics has been applied to detect biomarkers that can distinguish acute anterior uveitis (AAU) progression and treatment response (Guo et al., 2014). It allows determination of the metabolic pattern of the gut microbiota and discrimination between metabolites that may be associated with different disease courses (Peng et al., 2015; Huang et al., 2018; Vignoli et al., 2019). For example, host-bacterium cometabolites known to induce metabolic deteriorations in genetic and simple obesity in children determine specific bacterial genomes correlated with urine levels of these detrimental cometabolites (Heintz-Buschart et al., 2016).

Despite their costs and technical challenges, longitudinal and multiomic experimental designs are becoming indispensable for unravelling host-microbiome interactions during disease and for assessing causality in clinical microbiome investigations (Zhang et al., 2019). There are still some unsolved problems that interfere with the accuracy of the results, e.g., similar studies conducted by different groups of researchers sometimes yield varying results. The reason is that there is no standardization of sample preparation and analysis flow (Gorkiewicz et al., 2013; Xiong et al., 2015; Drago et al., 2016; Shobar et al., 2016; Nagata et al., 2019; Daliri et al., 2021). Importantly, a major challenge for metatranscriptomic sequencing is that it is difficult to eliminate rRNA contamination, as mRNAs only account for 5% of the total RNA in a cell (Liang et al., 2000; Jiang et al., 2016). Second, due to the limited richness of the comparison database, there are many measured results that are unable to match the function (Pasolli et al., 2019; Granata et al., 2020). Further work is needed to enrich the existing databases used for comparison.

Over the years, integrative multiomics analyses, referring to the combination of two or more omics analysis methods, have been proposed for studying host-microbiome interactions, which offer considerable information regarding microbial phylogeny and metabolic pathways and may provide clues for the specific proteins and metabolites that contribute to a specific host phenotype. (Daliri et al., 2021). Future investigations may consider combining several omic techniques rather than relying on only one or two techniques, which may be beneficial for compensating for the weakness of single-omic studies.

#### 2.2.2 Establishing a Well-Controlled Animal Experiment

Environmental and dietary factors exert tremendous impacts on microbiota compositions, rendering it difficult to rigorously confirm the cause-and-effect relationship between microbiota and disease in clinical studies. Recently, several novel experimental models have been established to study the roles of microbiota in health and disease in living animals.

##### 2.2.2.1 Germ-Free Animals and Antibiotic Usage

Removal of commensal microbiota by rearing animals in a germ-free (GF) environment or by oral antibiotic treatment has been used to explore the link between the gut microbiota and ocular diseases, especially autoimmune diseases. GF mice have been widely used in studies of allergies (Vercelli, 2021), conjunctivitis (Wang et al., 2018; Zaidi et al., 2018), autoimmune uveitis (Horai et al., 2015), and diseases with ocular manifestations, such as Sjögren syndrome (SS) (Trujillo-Vargas et al., 2020). However, a concern of using GF mice is that the normal development of the immune system is strongly dependent on the microbiome, such that under GF conditions, the animal’s response to immune stimuli may be blunted (Dzidic et al., 2018). Data from GF mouse studies must be interpreted in context, as several host physiologic parameters are altered in these mice. For example, GF mice have underdeveloped immune systems (Gaboriau-Routhiau et al., 2009; Ivanov et al., 2009; Atarashi et al., 2011), slower intestinal epithelial turnover (Savage et al., 1981), differences in epithelial gene expression (Chowdhury et al., 2007), and reduced body fat (Bäckhed et al., 2004).

The drawback of using broad-spectrum antibiotics is that there are unintended side effects on the host immune system. Compared with controls, mice treated with a cocktail of antibiotics have been observed to have weight loss and abnormal organs, such as reduced spleen size (Sun et al., 2021). This suggests that antibiotic usage leads to a duration of immune imbalance that must be considered in therapeutic strategies. Moreover, antibiotic cocktails do not completely exclude the direct or indirect effects of fungi or viruses on ocular disease.

Initially, a role for the gut microbiome in a phenotype of interest is determined by comparing antibiotic(ABX)-treated animals with untreated animals. If antibiotic treatment abrogates or exacerbates a model’s phenotype, it may support some undefined role for the gut microbiome. The underlying assumption is that the antibiotic does not possess an effect on the model’s phenotype itself. Thus, proper control groups, and often multiple different antibiotic regimens, are necessary to demonstrate a difference (Ericsson and Franklin, 2015). Another consideration is that even in the absence of overt changes in the compositions of the gut microbiome, antibiotics and other xenobiotics may significantly alter the transcriptional activity of the gut microbiome and impact the model’s phenotype (Maurice et al., 2013). The removal of even a small portion of commensal microbes by antibiotics might disrupt microbial syntrophy, which may not lead directly to phenotypic changes but may lead to subsequent changes in larger microbial populations, resulting in changed phenotypes. Thus, any alterations in phenotype by antibiotic treatment must be interpreted cautiously to separate the effects of the gut microbiome from the antibiotics themselves (Ericsson and Franklin, 2015).

##### 2.2.2.2 Methods of Humanized Gnotobiotic Models

To better simulate the human environment, the human microbiota has been applied to GF or ABX animals (Turnbaugh et al., 2009; Walter et al., 2020). Targeting fecal transplants at the strain level, a single causative organism may be identified to discuss causality more accurately. In the past decade, gnotobiotic human microbiota-associated (HMA) mice have been widely used to study causality and mechanisms of microbiome-disease associations, such as the contribution of a dysbiotic microbiome to a particular pathology (Walter et al., 2020). Compared to mice colonized with the microbiota from healthy controls, GF mice are usually colonized with the fecal microbiota from patients. A seminal study showed that GF transgenic mice expressing a myelin autoantigen-specific TCR reconstituted with fecal samples from MS patients developed more serious clinical scores on spontaneous EAE than healthy stool-recipient gnotobiotic mice (Berer et al., 2017).

While being an ideal model for studying the effects of the microbiome, there are some limitations of HMA mouse models that might affect data interpretations: (1) evolutionary considerations: under the pressure of long-term coevolution, the dynamic host-microbiota interactions might shift to a more host-beneficial and host-specific form; (2) ecological considerations: some species that abundantly exist in the human gut microbiome might fail to efficiently colonize the mouse gut or expand in a fashion that does not mimic their physiological states in the human gut but rather establishes a microbiome signature resembling that of recipient mice (Arrieta et al., 2016). This is because recipient mice are subjected to different ecological factors and immune environments than human donors. Ecological factors such as diet, human behavior, human genotype, and human immunity that help shape the microbial signature in humans are no longer present or may manifest differently in recipient mice (Arrieta et al., 2016). The data generated using HMA mouse models, therefore, may not be able to be used to accurately interpret the pathophysiological relevance of microbial imbalance in humans. Better simulation models must be developed.

## 3 Role of Microbiota in Ocular Disease

### 3.1 Autoimmune Uveitis

Autoimmune uveitis is a heterogeneous collection of diseases characterized by intraocular inflammation and a major cause of blindness in humans (Jabs et al., 2005; Forrester et al., 2013). Both genetic and environmental factors impact the development of autoimmune uveitis (Kamada et al., 2013). Based on current perspectives, its pathogenesis may be attributable to the abnormal activation of Th cells, specifically the imbalance between inflammatory Th1/Th17 and Treg cells (Eisenstein and Williams, 2009; Lee et al., 2014; Noack and Miossec, 2014). It is hypothesized that the activation of intraocular inflammation requires activated retinal antigen-specific lymphocytes to breach the blood-retinal barrier and migrate into the retina (Caspi, 2010). This raises an intriguing question of how the retinal antigens that are sequestered in the eye trigger T cell activation in peripheral circulating blood. Recently, emerging evidence has shown that gut microbiota may play an essential role in the development of uveitis.

Clinically, dysbiosis in gut bacterial communities has been observed in uveitis patients (Kalyana Chakravarthy et al., 2018). Transfer of the gut microbiome from patients with BD and VKHD, both of which are characterized by multiorgan inflammation involving uveitis, has been shown to significantly exacerbate disease severity in recipient experimental autoimmune uveitis (EAU) mice (Ye et al., 2018; Ye et al., 2020). The classic EAU animal model is induced by active immunization with the retinal protein interphotoreceptor retinoid-binding protein (IRBP) emulsified in complete Freund’s adjuvant (CFA) with heat-killed *Mycobacterium tuberculosis* (MTB), and severe ocular inflammation is observed within 2 weeks after induction (Weinstein and Pepple, 2018). Oral antibiotics substantially attenuate ocular inflammation in EAU mice (Nakamura et al., 2016). Notably, antibiotics applied through the intraperitoneal route have limited effects on eliminating commensals and do not alter disease progression in EAU, suggesting that the amelioration of EAU by antibiotic treatment is not due to the anti-inflammatory effects of antibiotics themselves (Nakamura et al., 2016). In another study, combining remodeling of the gut microbiome with immunosuppressive therapy significantly hindered the progression of EAU after inflammation onset (Zhou et al., 2020). The microbiome of EAU mice is distinct from that of controls (Horai and Caspi, 2019), which could be a result of robust immune activation by CFA.

To rule out the influence of CFA, spontaneous uveitis models have been introduced. These models were initially used to study how peripheral T cells enter the eye and later were used to study how endogenous antigens activate immune cells. The pathogenic mechanisms of spontaneous and immunized models are different because uveitis in spontaneous models is likely triggered by endogenous antigens (Horai and Caspi, 2019). R161H mice overexpress IRBP-specific TCRs on uveitis-susceptible B10 cells. The RIII background has been shown to expand the peripheral population of uveitis-relevant CD4^+^ T cells that appear in the intestinal lamina propria (LP) as early as 17 days of age, and uveitis has been shown to have 100% penetrance in these mice by 2 months of age (Horai et al., 2013). In one study, the degree of intraocular inflammation was significantly attenuated accompanied by reduced populations of Th17 cells in the intestinal LP when the mice were given a cocktail of oral broad-spectrum antibiotics before birth or were reared under GF conditions, and disease development was restored after cohousing with specific pathogen-free (SPF) mice (Horai et al., 2015). Interestingly, in contrast, the microbiota seem to be dispensable in another spontaneous model, *i.e.*, in Aire-deficient (*Aire* ^-^/^-^) mice. The Aire protein has a critical role in the process of thymic negative selection of autoreactive T cells and prevents autoimmunity by promoting the deletion of potentially self-reactive thymocytes (Anderson et al., 2002; Anderson et al., 2005; Proekt et al., 2017). Similar inflammatory infiltration occurs in several tissues in SPF and GF *Aire* ^-^/^-^ mice, including the retina, lung, pancreas, and stomach (Gray et al., 2007). The same finding has been reported in *Aire* ^GW/+^ *Lyn* ^–/–^ double-mutant mice with spontaneous posterior uveitis in which broad-spectrum antibiotics had no effects in treating the disease (Proekt et al., 2016). Thus, eliminating the influence of the microbiota alone cannot completely exclude other unknown mimics that may be able to activate high-affinity retina-specific T cells in the thymus of *Aire* ^-^/^-^ or *Aire* ^GW/+^ *Lyn* ^–/–^ mice. Further investigations are needed to delineate these seemingly contradictory results.

In a study of R161H IRBP^-/-^ mice, they displayed no uveitis but had a similar high frequency of Th17 cells in the gut compared with that in IRBP-sufficient R161H mice (Horai et al., 2015). Furthermore, R161H T cells cocultured with bacteria-rich intestinal contents *in vitro* can be turned into uveitis-pathogenic T cells, while this transformation was destroyed when intestinal contents were inactivated or were isolated from GF mice (Horai et al., 2015). These observations implied that IRBP is not the only antigen for uveitis and that microbiota stimuli can work as antigen mimics to activate T cells. Moreover, some studies have indicated that microbe metabolites can also affect uveitis. Propionate, one of the SCFAs produced by gut bacteria, has been confirmed to increase Tregs in the intestinal LP at the early stage of EAU in C57BL/6 mice, promoting barrier functions and maintaining the structural stability of the intestine (Smith et al., 2013; Nakamura et al., 2017; Sun et al., 2017). Thus, it has been proposed that intestinal commensal microbes, as antigenic mimics, alter the immune system, allow peripheral T cells to breach the blood retinal barrier, and cause inflammation in the eye.

In humans, anterior uveitis accounts for 85% of all instances of uveitis, representing the most common type (Gritz and Wong, 2004). Approximately 50% of patients with AAU are human leukocyte antigen B27 (HLA B27)-positive (Rosenbaum, 1989). HLA genes, such as major histocompatibility complex (MHC) genes, are the most polymorphic genes known (Jin and Wang, 2003). They are important mediators of the immune recognition of foreign pathogens by cytotoxic T cells and are therefore crucial in defense against foreign pathogens (Rosenbaum and Asquith, 2018). HLA B27 has been studied extensively for detecting the association between ankylosing spondylitis (AS) and spondyloarthropathies (SpA) (Khan, 2008). Recently, a well-defined clinical phenotype for SpA patients with AAU complications has been reported (Yang et al., 2018). However, HLA B27 transgenic animals develop a disease that strongly resembles human SpA but not AAU (Baggia et al., 1997). Interestingly, GF HLA-B27 rats do not display inflammation in the gut and joint, suggesting that commensals are probably involved to some extent in HLA B27-associated inflammation (Taurog et al., 1994).

To date, there are only limited investigations devoted to the immunopathogenesis of HLA B27-positive AAU. Testing fecal samples of AAU patients and healthy humans revealed no difference in gut microbiota composition; however, there was a clear difference in the fecal metabolic phenotypes (Huang et al., 2018). The absence of differences in microbiota may be due to a lack of sufficient patient samples. In some other HLA-associated diseases, the genotypes seem to affect the gut microbiome compositions. The HLA-DQ2 genotype that predisposes infants to celiac disease alters the gut microbiome, as demonstrated in a study of infants (Olivares et al., 2015). Further studies of the relationship between microorganisms and HLA may help us speculate on the pathogenesis of HLA-associated AAU. It has been shown that a large number of microbial peptides bind the HLA B27 molecule, and such microbial peptides may induce immune responses in target organs such as the eye and joint (Bodis et al., 2018). *Chlamydia*-derived peptides have been identified to immunize rats with occasional uveitis (Wakefield et al., 1991). Patients with AS have been described as having increased gut permeability (Colmegna et al., 2004). Additionally, endotoxin, which is a component of the gram-negative bacterial cell wall, has been used to induce anterior uveitis in experimental animal models (Hoekzema et al., 1992). In line with this evidence, the expression of Toll-like receptor 4, which binds endotoxin, has been found to be upregulated in the peripheral blood of AAU patients (Chang et al., 2007). One of the earliest immunological changes in HLA B27 transgenic rats with SpA is an upregulation of antimicrobial peptides such as S100A8 (calprotectin) in the colon (Asquith et al., 2016), which has been reported to be a biomarker for posterior uveitis (Olson et al., 1996). Notably, these changes may precede the development of dysbiosis in these animals (Rosenbaum and Asquith, 2018). These findings provide strong evidence for gut microbes or their metabolites as a trigger for altering the permeability of the gut wall and even entering the regional lymph nodes to generate a systemic immune response.

### 3.2 Age-Related Macular Degeneration

Age-related macular degeneration is a degenerative disorder that leads to impaired central vision, preferentially affecting the macular region of the retina (Chew et al., 2015). It is a polygenic disease that can be classified into dry and wet forms, both of which are influenced by environmental factors. These factors include disruptions in lipid, carotenoid, and inflammatory pathways induced by diet, smoking, and certain gene variants (Korobelnik et al., 2015; Chapman et al., 2019). Dry AMD causes damage to the retinal pigment epithelium (RPE), leading to indirect photoreceptor cell damage, while wet AMD is usually observed with choroidal neovascularization (CNV), which leads to RPE detachment and ultimately RPE cell death (Tadayoni, 2014).

A study reported that intestinal dysbiosis might occur in individuals with advanced AMD who had different types of gut bacteria compared to those in healthy older adults, such as the bacterial genera *Anaerotruncus* and *Oscillibacter* and the species *Ruminococcus torques* and *Eubacterium ventriosum* (Zinkernagel et al., 2017). However, the abundance of *Oscillospira, Blautia*, and *Dorea* was reduced in AMD subjects compared with healthy controls (Lin, 2018; Lin, 2019). Using high-throughput RNA sequencing in GF mice, Skondra and colleagues revealed key aspects of retinal gene regulation in AMD that are modulated by the intestinal microbiota (Nadeem et al., 2020). Although the exact pathogenesis of AMD remains poorly understood, certain inflammatory mechanisms associated with innate immunity have been identified. There are currently two hypotheses about microbial disorders influencing the progression of AMD, including immune regulation and promotion of nutrient absorption.

Regulated immune activation *via* recruitment of microglia and macrophages into the subretinal and choroidal areas, mast cell activation, and RPE immune activation presumably play a role in AMD pathogenesis (Handa et al., 2019). The gut microbiome may influence inflammation and the function of microglia during AMD progression. A study demonstrated that compared to normal-diet-fed mice, mice fed a high-fat diet were associated with intestinal dysbiosis and had a twofold increase in the number of microglia and macrophages within the local CNV lesions and that this increase was mitigated by eradicating the gut microbiota *via* neomycin application (Andriessen et al., 2016). Feeding aged mice heat-killed *Lactobacillus paracasei*, a lactic acid-producing bacterium, activated macrophages but paradoxically suppressed inflammation, resulting in a reversal of the deleterious *Firmicutes*/*Bacteroidetes* ratio and a decrease in both age-related inflammation and ganglion cell loss in the retina (Morita et al., 2018).

Oral supplements, antioxidants, minerals, and a diet containing carotenoids have been found in the Age-Related Eye Disease Studies (AREDS and AREDS2) to reduce the risk of AMD progression (Chew et al., 2015). Oral antioxidant supplementation according to AREDS2 is the only intervention that appears to slow the progression of AMD (Napolitano et al., 2021). Recent studies have shown that their efficacy is mediated by intestinal microbiota. For example, in a murine model of choroidal neovascularization, feeding mice a high-fat diet after administration of oral antibiotics can reduce CNV growth and improve alterations in the composition of the microbiome (Andriessen et al., 2016). Similar alterations in the intestinal microbiota have been shown to occur in mice with AMD-like characteristics fed a high-sugar diet, a high-fat diet or long-chain fatty acids (Zhang M. et al., 2018; Baim et al., 2019). On the other hand, a low-sugar diet did not impact the retina or the intestinal microbiota in this manner (Rowan et al., 2017). Although different lines of evidence indicate an association between AMD and diet-induced changes in gut microbiota, it is still unclear whether a causal relation truly does exist.

### 3.3 Glaucoma

Glaucoma is an incurable neurodegenerative disorder and the second leading cause of blindness worldwide; it is characterized by the death or dysfunction of retinal ganglion cells (RGCs), the axons of which lose intrinsic capacity to regenerate (Quigley and Broman, 2006; Lobato, 2008; Baden et al., 2016). Common therapeutic strategies to support visual restoration include protecting RGCs from degeneration, promoting regeneration of RGCs and axons after injury, and reestablishing the correct projection relationship (Hasegawa et al., 2004; Danesh-Meyer, 2011; Lusthaus and Goldberg, 2019; Schuster et al., 2020). Elevated intraocular pressure (IOP) is the most prominent threatened factor, while the inflammatory signals caused by injury recruit cellular and molecular processes and have been shown to affect the inherent regeneration ability (Yuan et al., 2021). Given the involvement of CD4^+^ T cells as well as the microbiota in the pathogenesis of glaucoma, it has been recently suggested that glaucoma should be included in the spectrum of autoimmune diseases (Geyer and Levo, 2020). An interesting study conducted by Chen et al. (2018) reported that a transient increase in IOP can induce autoreactive T cells to infiltrate the retina and that these T cells are presensitized by the symbiotic microbiota. Recently, commensal microbiota dysbiosis, including gut and oral microbiota dysbiosis, has been recognized to play a crucial role in the onset of neurological disorders and progressive neuronal loss (Schloss and Handelsman, 2004). DBA/2J mice are frequently used as a murine model for glaucoma because they spontaneously develop high IOP, mild intraocular inflammation, and glaucoma by 6–8 months of age (Libby et al., 2005). Interestingly, GF DBA/2J mice do not show typical axonal degeneration or neuronal loss at 12 months of age (Chen et al., 2018).

In the past two decades, the relationship between *Helicobacter pylori* infection and the pathogenesis of open-angle glaucoma (OAG) has become an active area of research. The connection between them was first proposed by Dr. Kountouras et al. (2001). Further study revealed that *H. pylori* might be a trigger of the systemic autoimmune response, which leads to the release of numerous inflammatory substances (Chmiela and Michetti, 2006). Mechanistically, *H. pylori* might influence the trabecular meshwork cell apoptotic process and crossreact with ciliary body epithelial antigens (Galloway et al., 2003; Burucoa and Axon, 2017). However, whether *H. pylori* plays a role in early onset is still a question (Deshpande et al., 2008; Kurtz et al., 2008). A meta-analysis combining all the relative results was conducted in 2015 (Zeng et al., 2015). The summary odds ratio (OR) and 95% confidence interval (CI) were calculated using the random-effects model. The results of this meta-analysis suggested a statistically significant association between *H. pylori* infection and primary open-angle glaucoma (POAG) or normal tension glaucoma (Zeng et al., 2015).

The potential correlation between the gut microbiota and glaucoma has been shown in several studies. POAG patients and healthy people have distinct profiles of gut microbiome compositions and serum metabolites (Gong et al., 2020). Butyrate, a gut bacterial metabolite, has been shown to lower IOP independently of blood pressure changes in rats (Skrzypecki et al., 2018). In addition, an increased level of trimethylamine, a uremic toxin produced by gut microbiota, has been observed in the aqueous humor of patients with glaucoma (Skrzypecki et al., 2021).

Heat shock proteins (HSPs) are highly conserved from bacteria to humans. Chen et al. (2018) demonstrated that glaucomatous mice induced by IOP may harbor HSP-specific memory T cells that were originally induced by commensal bacteria (Flemming, 2018). HSP27 and HSP60 have been found to be upregulated on RGCs in human glaucoma, and higher levels of serum HSP27-specific antibodies have been found in an experimental model (Chen et al., 2018). Low-dose subcutaneous lipopolysaccharide (LPS) administered to two animal models of glaucoma resulted in upregulation of TLR4 signaling and complement and activation of microglia in the retina and optic nerve (Astafurov et al., 2014). Enhanced oral bacterial loads have been shown to be correlated with a higher chance of glaucoma (Astafurov et al., 2014). Taken together, these results suggest that gut bacteria-triggered CD4^+^ T cells play a role in the induction of glaucoma by entering the eyes after the blood–retina barrier is damaged by pressure and causing neurodegeneration through crossreaction with HSP-expressing RGCs (Flemming, 2018). The exact role of gut microbiota in the development and progression of glaucoma remains to be explored.

### 3.4 Ocular Surface Microbiome Dysbiosis and Ocular Diseases

As the ocular surface is subjected to constant washing by tears and other antimicrobial ocular secretions, the presence of commensal bacteria at the ocular surface has been debated. However, cultural and sequencing evidence from ocular surface swabs clearly demonstrate the habitats of microbes on the ocular surface (Wu et al., 2003; Miller and Iovieno, 2009; Zhou et al., 2014; Huang et al., 2016). The ocular microbiota can be influenced by environmental conditions, disease states, use of antibiotics, age, sex, personal habits, contact lens wear, etc. (Stapleton et al., 2007; Lu and Liu, 2016; Ozkan et al., 2017; Wen et al., 2017; Cavuoto et al., 2018). Recently, persistent colonization of *E. faecalis* was identified on the ocular of patients with chronic ocular surface diseases (Todokoro et al., 2018). Alterations of the normal ocular microbial flora are related to several disease states, such as blepharitis, conjunctivitis, keratitis, trachoma, and dry eye syndrome (Schabereiter-Gurtner et al., 2001; Graham et al., 2007; Lee et al., 2012; Kugadas et al., 2016).

Microbial keratitis is an infectious ocular disease in which the cornea is inflamed. Under severe conditions, keratitis can lead to significant loss of vision and enucleation of the eye (Cruz et al., 1998). *Pseudomonas aeruginosa* (*P. aeruginosa*) is one of the gram-negative bacteria most frequently isolated from patients with bacterial keratitis (Al-Mujaini et al., 2009). The contribution of the ocular surface microbiome to the induction of keratitis by infectious *P. aeruginosa* has been documented (Kugadas et al., 2017). Wearing contact lenses is a possible risk factor for the development of microbial keratitis and other inflammatory eye conditions (Eguchi et al., 2017b; Miyazaki et al., 2020).

*Bacilli* are most prominent in patients with dry eye disease (Zilliox et al., 2020; Andersson et al., 2021). Meibomian gland dysfunction often leads to evaporative dry eye syndrome. The severity of MGD has been shown to be positively correlated with a higher isolation rate, a greater number of bacterial species, and a higher grade of bacterial severity (Jiang et al., 2018). *Staphylococcus*, *Corynebacterium*, and *Sphingomonas* may be involved in the pathophysiology of MGD (Pinna et al., 2005; Dong et al., 2019). Moreover, SS is a chronic autoimmune disease associated with dry mucosal surfaces and other systemic muscular pain (Both et al., 2017; Kuklinski and Asbell, 2017). SS-related dry eye patients share similar gut dysbiosis features (Kuklinski and Asbell, 2017). Several gut bacterial species are associated with the severity of dry eye parameters (de Paiva et al., 2016; Mendez et al., 2020; Moon et al., 2020).

Trachoma, caused by *Chlamydia trachomatis*, remains the leading infectious cause of blindness worldwide. A decrease in diversity and an increase in the abundance of *Corynebacterium* and *Streptococcus* were seen in participants with conjunctival scarring compared to normal controls. Importantly, *Chlamydia trachomatis* infections have been associated with reduced bacterial diversity and with an increase in the *Corynebacterium* and *Streptococcus genera* (Zhou et al., 2014). Pickering et al. reasoned that the potential mechanism may be associated with altered innate immune responses to the microbiota, dominated by altered mucin expression and increased matrix adhesion (Pickering et al., 2019). Moreover, *Corynebacterium mastitis* firmly colonized the conjunctiva increased its resistance to pathogens and stimulated the production of interleukin-17 by conjunctival T cells, allowing the recruitment of a greater number of neutrophils (St Leger et al., 2017).

Some studies have also reported the contribution of microorganisms to the development of ocular neoplasms. Examples include human papillomavirus, the cause of human conjunctival papilloma, which has been associated with squamous cell carcinoma, and HIV viruses, which have been associated with conjunctival squamous cell carcinoma (Miller and Iovieno, 2009).

### 3.5 Gut-Eye Axis

**Figure 1:**
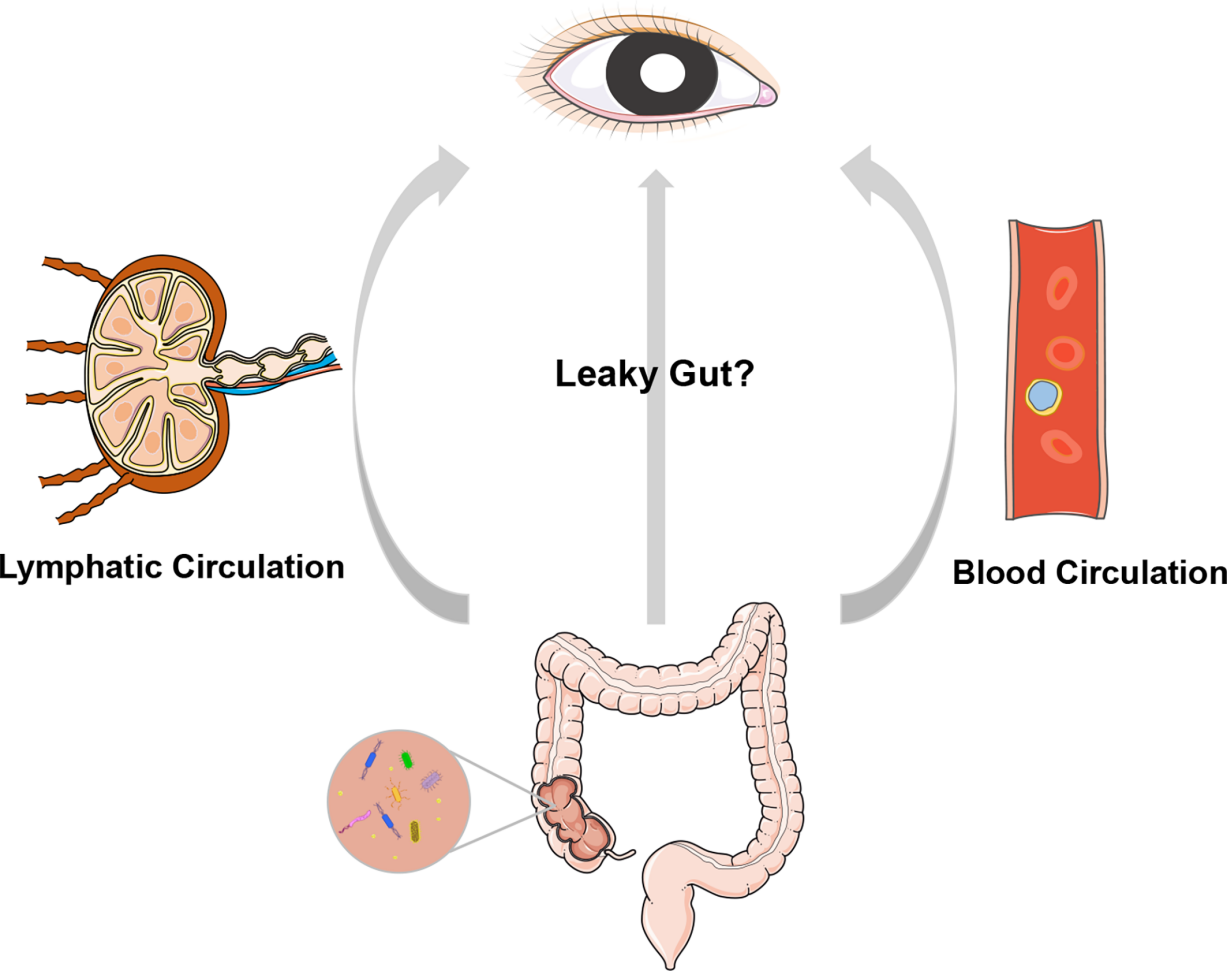
A cartoon illustration of the “gut-eye axis”. Dysbiosis of the intestinal microbiota of disruption in the intestinal barrier may result in translocation of the intestinal bacteria and/or their metabolites into circulatory system and lymphatic system, and further impact the eye, which is remotely located from gut.

A dogma in ophthalmic research is that the intraocular environment is always sterile under physiological conditions. Nevertheless, emerging evidence, including the translocation of intestinal bacteria into the circulating blood (Kell et al., 2015), liver (Vieira et al., 2018), pancreas (Aykut et al., 2019) and many other organs, argues against intraocular sterility. As the first and foremost finding, our group identified the presence of an intraocular microbiota *via* quantitative PCR, negative staining transmission electron microscopy, direct culture, and high-throughput sequencing technologies. We also analyzed aqueous humor samples collected from four species, i.e., rat, rabbit, pig, and macaque, and confirmed the existence of an intraocular microbiota (Deng et al., 2021). These observations raise an intriguing question of how microbes enter the eye (**Figure 1**). One speculation is that intraocular microbes originate from the gut, where they breach into circulating blood when a certain disease circumstance causes damage to the mucosal barriers and increases intestinal permeability. Both gut microbiota and their metabolites might be endogenous culprits of ocular diseases, possibly exerting their function by molecular mimicry and integrated immunological pathways. There is evidence that certain bacteria can survive in autophagosomes within phagocytes, à la a Trojan horse, and migrate with these cells through barriers from the gut to other body sites (Berthelot et al., 2015; O'Keeffe et al., 2015).

The immune response triggered by antigenic mimicry is the process whereby autoreactive T cells are generated through cross reactivity with gut microbial peptides as self-antigens (Rojas et al., 2018). In line with this theory, a study found that several peptides derived from commensal bacteria activated Ro60-reactive T cell hybridomas (Szymula et al., 2014). Ro60/SSA is one of the major autoantigens in SS and systemic lupus erythematosus. Repeated injections of the outer membrane protein A of *E. coli* induced the production of SS-related autoantibodies (Yanagisawa et al., 2018). Microbial cross reactivity has also been demonstrated in patients with systemic lupus erythematosus. One of these identified commensals, *Propionibacterium propionicum*, activated Ro60-specific memory CD4 T cells isolated from lupus patients (Greiling et al., 2018). Similarly, gut commensal bacteria have been shown to share homologous autoantigens in rheumatoid arthritis patients (Pianta et al., 2017).

Disruption in the intestinal barrier may result in leakage of bacteria or their metabolites into the blood or lymphatic system, irritating local or systemic immune responses. One such example is that SCFAs have been shown to increase colonic Treg frequencies in the gut in mice (Smith et al., 2013). In an EAU model, intestinal dysbiosis accompanies a disruption in intestinal integrity (Janowitz et al., 2019). In addition, oral administration of SCFAs can alleviate the development of EAU (Nakamura et al., 2017). The mechanism currently speculated is that bacterial products and pathogen-associated molecular patterns (PAMPS) enter the circulation and interact with downstream pattern recognition receptors (PRRs). The interaction between microbiota and Toll-like receptors (TLRs) activates the innate immune response. In cancer research, tumor-type-specific intracellular bacteria have been detected in multiple human tumors, although at low biomass (Nejman et al., 2020; Poore et al., 2020). These microbes are believed to play roles in tumorigenesis. Considerable evidence demonstrates the roles of microbes in Alzheimer’s disease, Parkinson’s disease, and autism, corroborating the theory of the gut microbiota-brain axis (Caputi and Giron, 2018; Angelucci et al., 2019; Morais et al., 2021). Likewise, maintaining homeostasis of the gut microbiota-eye axis might be a feasible strategy for the therapy of ophthalmic diseases.

## 4 Microbial Therapeutics

Ophthalmic antibiotics have been used to treat and prevent a variety of infectious and inflammatory conditions. The shift in the ocular microbiome influences ocular homeostasis and may increase the risk of eye infection (Miller and Iovieno, 2009; Mshangila et al., 2013). The administration of antibiotics causes ocular microbiota alteration, and extensive usage may promote the development of antibiotic resistance. Fecal microbiota transplantation (FMT), which involves placing stool from a normal donor into the gastrointestinal tract of a patient, has been controversial due to its safety and transient effect (Rosenbaum and Asquith, 2018). In addition, the challenge is the complexity of the diet-regulated gut microbiome, which makes it very difficult to identify the components that need to be added or subtracted for sustained benefit (Rosenbaum and Asquith, 2018). Probiotic and relative metabolite regimens and bacteriophage therapy have been proposed in recent years as surrogates for antibiotic treatment.

### 4.1 Probiotics and Relative Metabolites

Probiotic regimens have become a popular dietary intervention taking advantage of their transient and noninvasive properties, which help maintain a healthy immune system by improving gut health. Probiotics are thought to modulate immune responses, protect against physiological stress, suppress invasion of pathogens, modulate microbiota, and improve the barrier function of the gut epithelium (Shakya et al., 2019). *Lactobacilli*, for example, is able to decrease the number of neutrophil extracellular traps (Klenkler and Sheardown, 2004). *Bacillus fragilis* provides protective effects against autoimmune disease through its polysaccharide capsule (Knop and Knop, 2007). Some ongoing studies are evaluating the stability of an eye drop probiotic formulation containing *Saccharomyces boulardii* and *Lactobacillus rhamnosus* in patients with vernal keratoconjunctivitis. *Bifidobacterium* promotes the isolation and utilization of SCFAs, which have been identified to have a direct effect on modulating gut mucosal immunity and decreasing the severity of EAU (Knop and Knop, 2007; Rivière et al., 2016). The combination of probiotics including lactobacillus gasseri KS-13, Bifidobacterium bifidum G9-1, and B. longum MM-2 improved rhinoconjunctivitis during allergy through increasing serum total IgE and the percentage of Tregs from baseline (Dennis-Wall et al., 2017). Chisari et al. proved that the administration of *Enterococcus faecium* and *Saccharomyces boulardii* on the tear film was effective in ameliorating dry eye syndrome (Chisari et al., 2017) Changes in the gut microbiome after IRT5 treatment, a mixture of five probiotic strains, have been shown to improve clinical manifestations in the autoimmune dry eye model *via* the downregulation of antigen-presenting processes in immune networks (Choi et al., 2020). Meanwhile, IRT5 probiotics can modulate EAU by decreasing the percentage of pathogenic CD8+ T cells in drainage lymph nodes (Kim et al., 2017). Interestingly, *Lactobacillus paracasei* KW3110 improved the function of cultured RPE cells under chronic inflammatory stress and could relieve eye fatigue in humans safely (Yamazaki et al., 2020).

The selective metabolization of prebiotics in the gut by host microorganisms can purportedly determine the growth and functionality of local bacteria that influence human health (Napolitano et al., 2021). How probiotics modulate the intestinal microbial balance is poorly understood, and the molecular mechanisms have yet to be studied.

### 4.2 Bacteriophage Therapy

Some diseases have been shown to be associated with specific microbiota markers in recent studies. The precise modulation of microbiota *via* phage therapy shows potential for treating microbiota-associated diseases. Compared with traditional antibiotic therapy, phages display extremely high selectivity to target bacteria and good biosafety in humans. Phage-mediated modulation of microbiota has been shown to eliminate drug-resistant infections and improve conventional antitumor strategies (Zhang et al., 2021). Furthermore, it is a more efficient method because of its destructiveness to host bacteria and high fertility (Weinbauer, 2004).

Santos et al. reported that the isolated bacteriophages P2S2 and P5U5 were potential candidates for phage treatment of infection of ulcerative keratitis caused by *P. aeruginosa* in dogs (Santos et al., 2011). The therapeutic potential of Ply187AN-KSH3b, a chimeric phage endolysin derived from the Ply187 prophage, has been evaluated in treating bacterial endophthalmitis with promising results (Singh et al., 2014). Wang et al. (2016) used 4 phages selected from sewage to selectively kill cytolytic *Enterococcus faecalis* to treat ethanol-induced liver injury and steatosis in GF mice that were transplanted with the fecal microbiota of cytolysin-positive patients. A later study found that combining silver nanoparticles with M13 phage could specifically eliminate *Fusobacterium nucleatum* in the gut and reduce the number of immunosuppressive myeloid-derived suppressor cells, which improved the efficacy of checkpoint inhibitor-mediated immunotherapy for colorectal cancer (Dong et al., 2020). An advantage of using phages is that their effects on the microbiome are holistic, not limited to merely the removal of sensitive bacteria, and are cascadic on other bacterial species *via* the interaction between different bacteria (Wang et al., 2016; Hsu et al., 2019). Filamentous phages and T4 phage have been shown to have immunosuppressive effects in EAE or murine collagen-induced arthritis models (Rakover et al., 2010; Międzybrodzki et al., 2017). Recently, it was reported that after subconjunctival injection of the thermodynamically and chemically stable RNA nanoparticles derived from the three-way junction of the pRNA from bacteriophage phi29 DNA packaging motor, up to 70% of the retinal cells contained the nanoparticles at 24 h after the injection (Shi et al., 2018). This may provide a new way for intraocular drug delivery. Although the host microbiota has been proven to be involved in the occurrence and development of many diseases, most mechanisms of action between the microbiota and host are still not clear. The correlations between phages, microbiota, and hosts need to be further confirmed in the future (Zhang et al., 2021).

## 5 Conclusion

The gut microbiota is so important that it dictates normal development and homeostasis in mammals, which may subsequently affect ocular health. Although a growing amount of evidence suggests that ocular disease progression is associated with altered gut microbial composition, the direct interconnections between gut microbiota and eye function require a solid molecular mechanistic foundation. We have recently identified an intraocular microbiota in normal living animals and in patients with ocular diseases (Deng et al., 2021). These observations raise several interesting questions: How did the microbes enter the eye and affect the ocular diseases? Did they originate from the gut microbiota and travel through the peripheral circulating blood? How do intestinal and intraocular microbiota crosstalk with each other? Microbes has been recently reported to present in normal human circulating blood (Damgaard et al., 2015; Potgieter et al., 2015; Paisse et al., 2016; Schierwagen et al., 2019). One hypothesis is that temporary breach of blood-retina barrier gives the bloodstream microbes which presumably emanate from the gastrointestinal tract a chance to enter the eye. Migration of microorganisms into the eye may be mediated by phagocytes under the immune imbalance caused by ocular diseases. Detailed mechanisms relating to the potential immunological links between microbes-derived antigens and ocular tissues has been discussed in detailed in another review (Li et al., 2020). The process likely involves TLRs, resident ocular antigen-presenting cells, such as ocular dendritic cells, MHCs and miscellaneous inflammatory mediators. The interrelationship between microbiota and homeostatic immunity of whole body (such as abundance commensals of oral, lungs and skin) as well as of local ocular tissues warrants further investigations, which may provide insights into targeting microbiota as a therapeutic strategy for ocular diseases and other diseases associated with gut microbiota in a similar paradigm.

## Author Contributions

WX read the literature related to the topic and participated in drafting the manuscript. JL participated in searching literature and revising the manuscript. YZ and BZ prepared figures. LW participated in the design, revision, and final approval of the manuscript. All authors read and approved the final manuscript.

## Conflict of Interest

The authors declare that the research was conducted in the absence of any commercial or financial relationships that could be construed as a potential conflict of interest.

## Publisher’s Note

All claims expressed in this article are solely those of the authors and do not necessarily represent those of their affiliated organizations, or those of the publisher, the editors and the reviewers. Any product that may be evaluated in this article, or claim that may be made by its manufacturer, is not guaranteed or endorsed by the publisher.
